# Associations between Social Capital and Self-Rated Health among Men Who Have Sex with Men in Japan

**DOI:** 10.3390/healthcare12100997

**Published:** 2024-05-13

**Authors:** Adam O. Hill, Noriyo Kaneko, Carl M. Page, Natalie Amos, Kohta Iwahashi, Adam Bourne, Stuart Gilmour

**Affiliations:** 1 Graduate School of Public Health, St Luke’s International University, Tokyo 104-0044, Japan; 2 Australian Research Centre in Sex, Health and Society, La Trobe University, Bundoora, VIC 3086, Australia; 3 School of Nursing, Nagoya City University, Nagoya 467-8601, Japan; 4 Graduate School of Social Systems, Kitakyushu University, Fukuoka 802-8577, Japan; 5 NPO AKTA, Tokyo 160-0022, Japan; 6 Kirby Institute, UNSW Sydney, Sydney, NSW 2052, Australia

**Keywords:** MSM, self-rated health, social capital, Japan

## Abstract

Men who have sex with men (MSM) are significantly more likely to report poor health compared to the general population in Japan and internationally. Social capital has been observed as an important component of positive health and well-being outcomes among MSM. However, there is limited research investigating how alter sexuality (possessors of actual resources embedded in social capital networks) mitigates health outcomes. In an online survey of 1564 MSM in Japan, we investigated social correlates of poor self-rated health among MSM, including MSM and heterosexual social networks. Multiple logistic regression revealed that poor health was associated with older age, lower education, and part-time and unemployment. Poor health was inversely correlated with bisexual behavior and high MSM or heterosexual social capital. In order to decrease health disparities among MSM in Japan, interventions focusing on increasing social capital among deprived groups, such as those with lower socio-economic status, older MSM, and those whose sex partners are exclusively male, may be effective.

## 1. Introduction

Men who have sex with men (MSM) have been shown to experience poorer health outcomes compared to the general male population across various contexts and studies globally [1,2,3]. This disparity stems from a complex interplay of behavioral, social, and systemic risk factors. Research has consistently identified higher rates of HIV and other sexually transmitted infections (STIs) among MSM [4], as well as a higher prevalence of mental health challenges [1,5], suicidal ideation and attempts [1,6], and problematic alcohol and other drug use [7,8], reflecting the impact of stigma, discrimination, and stress associated with their minoritized status [1,9,10]. These health disparities are further exacerbated by barriers to healthcare access, including fear of discrimination and lack of culturally competent care [11,12].

The Japanese situation mirrors the global context, with MSM disproportionately at risk of contracting HIV, despite the potential for its elimination [13], accounting for 78% of all Japanese male HIV cases in 2019 [14]. While there is no data at present regarding the self-rated health of Japanese MSM, studies have found they face high levels of sexuality-based bullying [15], harassment [16], stigma [17] and suicidal ideation [6]. Moreover, Japanese culture, emphasizing social conformity compared to many western countries, may particularly amplify the effects of stigma on MSM, making it more challenging for them to access social support and healthcare resources, which are crucial for positive health outcomes [11,15]. Conversely, the different religious and historical contexts of sexual diversity in Japan [18] may mitigate some of the effects of stigma and discrimination in Japan, especially among MSM who otherwise conform socially. The overall nature of these social support structures and their interaction with individual behavior is often summarized in the concept of social capital [19].

In this research, we conceptualize social capital as the range of resources that individuals may access via their social connections [19]. This concept encompasses three key components: the ‘ego’, which refers to the individual who taps into these resources through their social networks; the ‘alters’, who are the holders of these resources and provide them to the ego; and the resources themselves, which range from, for example, emotional support to a modest financial loan. Social capital is increasingly associated with better health outcomes. In the general population, high levels of social capital have been linked to healthier living, increased use of preventive health services, and better overall self-rated health, highlighting the importance of social networks and community engagement for health outcomes [20,21]. For MSM, social capital has been associated with emotional support, reduced feelings of isolation, and better access to health information and services, mitigating some of the adverse effects of stigma and discrimination on health [22]. For example, in Sweden, gay and bisexual men participating in a national survey had poorer self-rated health than the general population; however, when controlling for social capital, there was no longer a significant health disparity between them [23]. Social capital has also been found to be associated with higher self-rated health in Japan among the general population [24]. Although there is no data specifically regarding social capital and self-rated health among MSM in Japan, social capital was associated with a variety of health-positive outcomes, including lower rates of suicidal ideation, and higher levels of HIV testing and condom use in previous publications using data from the survey upon which this study is based [6,25]. Based on these findings, we hypothesize that MSM in Japan with higher levels of social capital will report better health outcomes compared to those with lower levels of social capital.

While there is strong evidence that we should clearly define the difference between individuals accessing resources (the ‘ego’) and those who provide these resources (the ‘alter’) in studies of social capital [20,26,27], this distinction is rarely made in research [28]. Furthermore, the effects derived from social capital are context-dependent. For example, MSM social capital networks were associated with increased uptake of HIV testing [25]. However, these same networks may also precipitate higher-risk behaviors due to peer influences [29], resulting in lower condom usage with sexual partners [25]. Conversely, in the same study, heterosexual social capital among MSM was correlated with increased condom use [25]. It is therefore essential to differentiate between social capital networks that influence health promotion and those that might promote health-detrimental behaviors.

Given the unique social and cultural context of Japan, it is critical to understand how social capital functions among Japanese MSM and how it can be leveraged to improve their health outcomes. Despite this, we have not identified any prior studies regarding correlates of health among MSM in Japan, nor any globally that stratify social capital networks by the sexual identity of the alter (MSM vs. heterosexual), particularly whether social capital networks accessed via MSM or heterosexual alters correlate with varying health outcomes. This study aims to fill this research gap. Findings from this research will identify correlates between health and social capital and could shape the development of social capital-driven health interventions to mitigate health disparities faced by MSM in Japan.

## 2. Materials and Methods

### 2.1. Survey

We conducted a cross-sectional survey investigating the correlation between social capital and various health behaviors among MSM utilizing gay mobile apps in the Greater Tokyo area. This survey employed an anonymous, self-administered, and structured questionnaire. Approval for the study was obtained from the Human Research Ethics Committee at the University of Melbourne (HREC 1646197).

The survey questionnaire was translated from English into Japanese by a native translator and then back-translated into English by an independent translator to ensure accuracy. Informed consent was obtained from all survey respondents, and MSM helplines and information services were provided to all participants who accessed the survey link. Participants had the option to receive the survey results via email and could also choose to enter a lottery for prizes of up to 80 USD in gift cards. A total of 1657 participants completed the survey. Duplicate IP addresses and email addresses were cross-checked and removed to address any instances of participants completing the survey multiple times. Additionally, checks were conducted to eliminate incomplete responses, resulting in a final sample of 1564 participants for analysis, all of whom completed the survey and questions pertaining to social capital.

### 2.2. Recruitment

The recruitment methodology employed in this study has been discussed in more detail in previous publications [30,31]. In summary, participants were recruited utilizing the geo-location feature inherent in gay mobile applications, mirroring methodologies previously utilized for the recruitment of Grindr users within the United States [32,33]. Initially, the mobile applications were accessed on a mobile phone, and participants were selected systematically, with one online user chosen from each row until 50 users who had not been previously contacted were identified. These individuals were subsequently messaged with a link redirecting them to the survey platform. Through this process, a total of 215 participants were successfully recruited between November 2016, and January 2017. Subsequently, in January 2017, a splash screen advertisement poster—an advertisement occupying the full screen upon app initiation—was deployed on gay mobile applications for one week, incorporating a hyperlink directing users to the survey platform. Notably, this recruitment approach had previously demonstrated efficacy in the United States [34,35]. The advertisement was displayed in rotation upon every application launch by users in the Greater Tokyo area, resulting in the recruitment of an additional 1442 participants.

### 2.3. Participants

Participants included individuals aged 18 years or older who self-identified as MSM (defined as identifying as homosexual, gay, or bisexual, or having sexual experiences with other men) and provided online consent to participate in the survey. Participation was entirely voluntary.

### 2.4. Measures

Socio-demographic questions were adapted from previous research conducted in Japan [36,37]. These encompassed inquiries regarding age, gender, sexual identification, marital status (note: marriage is legally recognized only between men and women in Japan), birthplace, current residence (categorized as Central Tokyo, Greater Tokyo, or other prefectures), self-rated health status, highest level of education attained, employment status, and the nature of their sexual relationships (solely with men or involving both men and women). Consistent with prior studies in Japan [38], self-rated health status was assessed using a Likert scale from 1 (very unhealthy) to 5 (very healthy). For analytical purposes, and in alignment with the methodology of previous research [38], this study categorized self-rated health into a binary variable. Ratings of 1 (very unhealthy), 2 (unhealthy), and 3 (neutral) were grouped as ‘Fair/Poor Health’, while 4 (healthy) and 5 (very healthy) were classified as ‘Healthy’.

In addition, we incorporated measures to assess engagement with the gay community. These measures asked participants whether they had ever attended a gay bar, event, or bathhouse (*hattenba*), or participated in organized gay group activities in the past six months. The responses to these questions were recorded as either ‘yes’ or ‘no’. 

Social capital was assessed using the Resource Generator, which is a culturally adaptable and validated instrument [39] designed to evaluate individual social capital. This concept of social capital is defined as the valuable resources individuals can access through their social networks [28]. The instrument comprised 18 questions specifically adapted to the Japanese context, which gauged participants’ ability to access physical and mental support resources within their social networks, whether composed of MSM or heterosexual individuals. An illustrative question from the instrument was, “Do you have access to an MSM or a heterosexual person whom you trust very much”? Response options included ‘heterosexual’, ‘MSM’, ‘both’, and ‘neither’. Scores for heterosexual and MSM social capital ranged from 0 to 18 points, respectively.

To differentiate between participants with varying levels of social capital, three groups (high, medium, and low social capital) were established based on the distribution of their scores. High social capital participants were identified as those scoring one standard deviation (SD) above the overall mean, while low social capital participants scored one SD below the mean. Participants falling between these extremes were categorized as medium social capital. The MSM social capital scale demonstrated high reliability (Cronbach’s alpha = 0.91), as did the heterosexual social capital scale (Cronbach’s alpha = 0.91).

### 2.5. Statistical Analysis

The analyses were performed using STATA version 16. Multivariable binary logistic regression analysis was employed to explore the correlates of fair or poor health. To investigate correlates of fair or poor health, univariable binary logistic regression analyses were initially conducted separately for all variables (e.g., socio-demographics or gay community participation). These variables served as predictor variables, while fair or poor health was treated as the outcome variable (dependent variable). Subsequently, multivariable binary logistic regression analyses were performed to assess independent associations between the predictor (independent) variables and the outcome (dependent) variable (i.e., fair or poor health), while adjusting for the effects of other predictor variables. Only variables that yielded *p*-values of 0.10 or less in the univariable analyses were considered eligible for inclusion in the respective multivariable model to assess their independent association with the self-rated health of MSM participants.

Adjusted odds ratios, along with their corresponding 95% confidence intervals and *p*-values, are reported where relevant. Additionally, variables within the multivariable model did not exhibit evidence of multicollinearity, as indicated by variance inflation factor (VIF) values ranging from 1.04 to 1.27 and tolerance values ranging from 0.83 to 0.93.

## 3. Results

### 3.1. Socio-Demographics and Gay Community Participation

Table 1 illustrates various demographic characteristics and attributes of the participants. Age distribution indicates that a significant portion of participants fell within the age groups of 26 to 35 years (33.6%) and 36 to 40 years (30.7%), with smaller proportions in the under 25 years (19.4%) and over 46 years (16.3%) categories. The majority of participants were born in Japan (96.4%), with a minority born in other countries (3.6%). Regarding location, the majority resided in Central Tokyo (53.6%), followed by Greater Tokyo (33.7%) and other prefectures (12.6%). Educational backgrounds vary, with notable percentages holding a university degree (48.3%) or high school diploma (25.2%). In terms of occupation, most participants were engaged in full-time work (68.2%), while smaller proportions were involved in part-time work (11.5%) or study (9.9%). The majority were unmarried (95.9%), and most reported having sex exclusively with men (91.2%). Attendance at gay bathhouses was divided, with 52.0% reporting no attendance and 48.0% reporting attendance. Additionally, a majority had attended gay bars or events (56.9%), while a smaller proportion had participated in gay group activities in the past six months (13.4%). Levels of MSM and heterosexual social capital vary, with notable percentages falling into the medium categories (MSM social capital: 59.3%, heterosexual social capital: 61.1%). Finally, regarding self-rated health, a notable proportion of participants reported fair or poor health (36.6%), while the majority indicated being healthy (63.4%).

### 3.2. Socio-Demographic Correlates of Fair or Poor Self-Rated Health—Multivariable Results

Based on the multivariable regression analysis (Table 2), the odds of fair or poor health among participants aged 26 to 35, 36 to 40, and over 46 were not statistically significant different to those under 25. Similarly, there was no significant difference in the odds of fair or poor health between participants born in Japan and those born in other countries. However, participants with a university degree (AOR = 0.50, 95% CI: 0.38–0.66, *p* < 0.001) and those with a graduate degree (AOR = 0.55, 95% CI: 0.36–0.85, *p* = 0.007) had about half the odds of fair or poor health compared to those with a high school education. Additionally, unemployed participants were approximately 2.61 times as likely to report fair or poor health (AOR = 2.61, 95% CI: 1.53–4.44, *p* < 0.001) compared to those in full-time work. Participants with both male and female partners were about 40% less likely to report fair or poor health (AOR = 0.60, 95% CI: 0.39–0.92, *p* = 0.019) compared to those with only male partners. 

### 3.3. Social Capital Correlates of Fair or Poor Self-Rated Health—Multivariable Results

Participants with high MSM social capital were approximately 43% less likely (AOR = 0.57, 95% CI: 0.39–0.82, *p* = 0.003) and those with high heterosexual social capital were about 52% less likely (AOR = 0.48, 95% CI: 0.32–0.72, *p* < 0.001) to report fair or poor health compared to those with low social capital in their respective categories.

## 4. Discussion

There was evidence that social capital plays a role in self-rated health for MSM in Japan, as reflected in several factors. Participants with a university degree or a graduate degree had lower odds of reporting fair or poor health compared to those with a high school education. Unemployed participants were more likely to report fair or poor health compared to those in full-time work. Participants with both male and female partners had lower odds of reporting fair or poor health compared to those with only male partners. The findings indicate that social capital emerges as the most robust predictor of good health among participants; participants with high MSM social capital and high heterosexual social capital had half the odds of reporting fair or poor health compared to those with low social capital in their respective categories. Moreover, the segregation of alter sexual identity enables us to see disparities in MSM and heterosexual social capital, such as how heterosexual social capital is more strongly associated with better health than MSM social capital. These findings underscore the importance of considering both heterosexual and MSM social capital separately when examining their associations with health outcomes, as they may have unique and potentially divergent impacts.

Previous analyses of this dataset have revealed a significant inverse correlation between medium and high levels of MSM social capital and recent suicidal ideation, resulting in reductions in odds by 40% and 60%, respectively [6]. Conversely, heterosexual social capital did not display a significant association in this regard [6]. However, in terms of self-rated health, our study uncovers significant associations between both MSM and heterosexual social capital and decreased odds of reporting poor or fair health status, with heterosexual social capital demonstrating stronger predictive capabilities. These differences are noteworthy, as prior investigations utilizing this dataset found that MSM social capital was linked to lower rates of condom use with regular partners, while heterosexual social capital was associated with higher condom use with casual partners [25], consistent with existing research on the relationship between social capital and HIV risk behaviors among MSM [22]. Given the heightened HIV risk among MSM compared to the general population in Japan [40,41,42,43], the perceived higher HIV risk within MSM social capital networks may adversely affect the self-perceived health status of MSM in Japan compared to those more closely connected to heterosexual social capital networks. Moreover, compared to heterosexual social capital, MSM social capital is primarily nurtured through gay dating apps and venues frequented by the gay community, often characterized by alcohol consumption and sexual encounters [44,45], potentially further impacting self-perceived health status through alcohol use and engagement in sexual activity. Nonetheless, it is important to highlight that high MSM social capital remains correlated with a two-fifths reduction in the likelihood of reporting poor or fair health. This association may be attributed to mechanisms akin to those observed in the reduction of suicidal ideation, where emotional support, particularly from ‘family of choice’, plays a pivotal role in promoting health and overall well-being [46]. In contrast, heterosexual social capital typically exhibits greater uniformity [47], being cultivated within familial and socioeconomically homogeneous circles such as educational and professional settings [47,48], thus fostering positive health norms and providing important health resources [49].

Other factors associated with poor or fair health included unemployment and lower educational attainment, as has frequently been observed in the general population globally [50]. However, it is understood that sociodemographic challenges, typically correlated with poor health outcomes in the broader population, are exacerbated among MSM due to minority stressors [9]. Sexual minority youth in Japan often encounter bullying and harassment, notably in educational [15] and occupational environments. Such adverse experiences can lead to reduced educational attainment and employment opportunities, subsequently diminishing levels of heterosexual social capital—which is equally if not more important for good health. Several factors could contribute to the difference in self-rated health between MSM who have sex with both men and women and those who have sex with only men in Japan, including stigma and discrimination against sexual minorities in Japan. MSM who have sex exclusively with men may experience higher levels of stigma and discrimination compared to those who have sex with both men and women. This increased stigma can negatively affect mental health and, consequently, self-rated health. Moreover, participants who have sexual relationships with both men and women might benefit from greater social acceptance in some contexts by ‘passing’ as heterosexual, as their behavior may align more closely with heteronormative expectations. This might not completely shield them from stigma but could lead to less direct discrimination, contributing to a better self-rated health perception. Additionally, the nature and structure of support networks may differ between these groups. MSM who engage in sex with both men and women may have access to a broader range of supportive relationships, both within and outside the gay community, potentially providing a buffer against stressors. 

Participation in organized activities within the gay community closely approached significant association with lower rates of poor or fair health in Japan, suggesting that MSM community initiatives conducted by non-governmental organizations (NGOs) and MSM community centers effectively reinforce health-promoting social norms among participants, consistent with prior research findings [51]. Given the limited funding allocated to gay community centers in Japan [52], there is a need for greater resources to facilitate the widespread dissemination of these health-promoting norms throughout social networks. It is therefore of utmost importance to fund initiatives aimed at engaging more MSM in sexual-minority-related community activities and NGOs in order to build social capital and disseminate the fostering and adoption of health-positive norms among Japanese MSM, while also working to address systemic societal issues such as reducing harassment and stigma towards HIV and sexual minorities in Japan. 

### Limitations

This study contributes valuable insights into the roles of alter sexuality and social capital in mitigating poor health outcomes in Japan’s MSM population, a field where research is notably sparse. Nevertheless, it is important to acknowledge the limitations inherent in this research. Data were sourced primarily from gay mobile applications, which may not encompass the full spectrum of the MSM demographic in Japan. Despite a sizeable sample and its novelty in assessing social capital among users of these applications—thereby including a segment often absent from previous research—the findings may not be wholly generalizable. Self-reported behaviors and attitudes could be influenced by social desirability bias, potentially leading participants to underreport behaviors they deem unfavorable, although the anonymous and online nature of the survey likely reduced this effect [53]. The tool used for measuring resources, the resource generator, is a validated instrument with a history of use in Japan [36,37]. Yet, given the lack of a standardized method for measuring social capital and the resultant challenges in comparing results across different studies [54,55], our conclusions should be interpreted with caution. Continued research is necessary to refine our understanding and measurement of social capital within this context. Moreover, it should be noted that the perceptions of health and the impacts of societal norms and stigma on individuals are highly complex and multifaceted. The explanations provided here are generalized and might not fully capture the specific dynamics at work within Japan. Detailed, culturally specific qualitative research would be beneficial to understand these phenomena fully within the Japanese context. 

## 5. Conclusions

This study highlights several critical determinants of self-rated health among MSM in Japan. Higher education and employment status are reaffirmed as protective factors against poor self-rated health, aligning with global trends. MSM with both male and female partners reported better health outcomes than those with exclusively male partners, suggesting that bisexual behavior may be associated with less stigma and more diverse social support in the Japanese context. Social capital emerged as the most significant health determinant; high levels within MSM and heterosexual networks were strongly predictive of better health, albeit with heterosexual social capital showing a more pronounced effect. This disparity may be attributable to the differing natures of these networks, with heterosexual social capital often formed in more stable and socially accepted environments. Our results reinforce the necessity of fostering robust sexual minority social networks through community engagement and gay non-profit organizations to mitigate health disparities faced by MSM in Japan. They also show that fostering an inclusive and non-discriminatory society, where people of differing sexual backgrounds can interact freely and build social capital together, is crucial for the health of sexual minorities, and Japan should continue its progress towards a fully equal society whose members can freely express and enjoy their sexuality without barriers, stigma or discrimination.

## Figures and Tables

**Table 1:** Socio-demographics (n = 1564).

	n	%
**Age**		
Under 25	302	19.4
26 to 35	523	33.6
36 to 40	478	30.7
Over 46	254	16.3
**Birth place**		
Japan	1507	96.4
Other	57	3.6
**Location**		
Central Tokyo	833	53.6
Greater Tokyo	524	33.7
Other prefecture	196	12.6
**Education level**		
High school	394	25.2
Two year uni	263	16.8
University	756	48.3
Graduate degree	151	9.7
**Occupation**		
Full-time work	1065	68.2
Part-time work	180	11.5
Student	155	9.9
Self-employed/freelance	95	6.1
Unemployed	67	4.3
**Marital status**		
No	1499	95.9
Yes	64	4.1
**Intercourse partner gender**		
Men only	1408	91.2
Both men and women	136	8.8
**Ever attended gay bathhouse**		
No	810	52
Yes	748	48
**Ever attended gay bar or event**		
Yes	890	56.9
No	674	43.1
**Participated in gay group activity in past 6 months**		
No	1351	86.6
Yes	209	13.4
**MSM Social Capital**		
Low MSM SC	325	20.8
Medium MSM SC	927	59.3
High MSM SC	312	20
**Heterosexual Social Capital**		
Low MSM SC	342	21.9
Medium MSM SC	956	61.1
High MSM SC	266	17
**Self-Rated Health**		
Fair or poor health	572	36.6
Healthy	990	63.4

**Table 2:** Multiple Regression Results—Correlates of Poor Health among MSM in Japan (n = 1564).

Variable	Unhealthy		OR (95% CI)	*p*-Value	AOR (95% CI)	*p*-Value
**Age**	n	%				
Under 25	86	28.5	REF			
26 to 35	180	34.6	1.39 (1.03–1.89)	0.032	1.19 (0.82–1.74)	0.361
36 to 40	197	41.2	1.84 (1.36–2.49)	0.000	1.39 (0.94–2.04)	0.096
Over 46	105	41.3	1.86 (1.32–2.63)	0.000	1.40 (0.91–2.16)	0.124
**Birth place**						
Japan	560	37.2	REF			
Other	12	21.1	0.46 (0.25–0.85)	0.013	0.68 (0.35–1.31)	0.248
**Residential location**						
Central Tokyo	301	36.1	REF			
Greater Tokyo	204	39.1	1.19 (0.95–1.48)	0.124		
Other prefecture	63	32.1	0.94 (0.68–1.29)	0.682		
**Education level**						
High school	188	47.7	REF			
Two year uni	112	42.8	0.86 (0.63–1.17)	0.325	0.78 (0.56–1.09)	0.152
University	228	30.2	0.47 (0.37–0.61)	0.000	0.50 (0.38–0.66)	0.000
Graduate degree	44	29.1	0.44 (0.29–0.65)	0.000	0.55 (0.36–0.85)	0.007
**Occupation**						
Full-time work	367	34.5	REF			
Part-time work	80	44.4	1.57 (1.15–2.15)	0.005	1.11 (0.78–1.57)	0.570
Student	38	24.5	0.58 (0.40–0.85)	0.005	0.78 (0.48–1.28)	0.324
Self-employed/freelance	44	46.3	1.64 (1.08–2.48)	0.020	1.73 (1.13–2.65)	0.011
Unemployed	42	62.7	3.19 (1.93–5.28)	0.000	2.61 (1.53–4.44)	0.000
**Marital status**						
No	552	36.9	REF			
Yes	20	31.8	0.73 (0.44–1.23)	0.241		
**Sex partner gender**						
Only men	533	37.9	REF			
Both men and women	34	25.0	0.50 (0.34–0.75)	0.001	0.60 (0.39–0.92)	0.019
**Ever attended gay bathhouse**						
No	292	36.1	REF			
Yes	278	37.2	1.09 (0.89–1.33)	0.406		
**Ever attended gay bar or event**						
No	259	38.4	REF			
Yes	313	35.3	0.89 (0.73–1.09)	0.274		
**Participated in gay group activity in past 6 months**						
No	509	37.7	REF			
Yes	63	30.1	0.70 (0.51–0.96)	0.025	0.73 (0.53–1.02)	0.066
**MSM Social Capital**						
Low MSM social capital	142	43.7	REF			
Medium MSM social capital	345	37.3	0.77 (0.59–0.99)	0.042	0.79 (0.60–1.05)	0.107
High MSM social capital	85	27.2	0.48 (0.35–0.67)	0.000	0.57 (0.39–0.82)	0.003
**Heterosexual Social Capital**						
low heterosexual social capital	162	47.4	REF			
Medium heterosexual social capital	353	37.0	0.65 (0.51–0.84)	0.001	0.80 (0.61–1.05)	0.113
High heterosexual social capital	57	21.4	0.30 (0.21–0.44)	0.000	0.48 (0.32–0.72)	0.000

## Data Availability

Data is available to readers upon reasonable request.
